# Influence of industry standard feeding frequencies on behavioral patterns and rumen and fecal bacterial communities in Holstein and Jersey cows

**DOI:** 10.1371/journal.pone.0248147

**Published:** 2021-03-05

**Authors:** Vanessa M. De La Guardia-Hidrogo, Henry A. Paz

**Affiliations:** Department of Animal and Dairy Sciences, Mississippi State University, Mississippi State, Mississippi, United States of America; INRAE, FRANCE

## Abstract

This study aimed to evaluate the effects of feeding frequency on behavioral patterns and on diurnal fermentation and bacteriome profiles of the rumen and feces in Holstein and Jersey cows. Ten Holstein and 10 Jersey cows were offered a TMR (53:47 forage-to-concentrate ratio dry matter basis) for *ad libitum* consumption and were randomly allocated within breed to one of the following feeding frequencies: (1) TMR delivered 1×/d (at 0600 h) or (2) TMR delivered 2×/d (at 0600 and 1800 h). The experiment lasted for 28 d with the first 14 d for cow adaptation to the Calan gates and the next 14 d for data collection. On d 23 and 24, an observer manually recorded the time budget (time spent lying, eating, drinking, standing, and milking), rumination activity, and number of visits to the feeding gate from each animal. On d 28, 5 concomitant collections of rumen and fecal samples were performed at intervals of 6 h via esophageal tubing and fecal grab, respectively. The bacteriome composition from these samples was determined through sequencing of the V4 region of the 16S rRNA gene. Feeding frequency did not affect behavioral patterns; however, Holstein cows spend more time lying (15.4 vs. 13.5 ± 0.8 h) and ruminating (401 vs. 331 ± 17.5 min) than Jersey cows. Fermentation profiles were similar by feeding frequency in both breeds. While no major diurnal fluctuations were observed in the fecal bacterial community from both breeds, diurnal fluctuations were identified in the rumen bacterial community from Holstein cows which appeared to follow pH responses. Overall, the bacterial community composition was not differentiated by industry standard feeding frequencies but was differentiated by breed and sample type.

## Introduction

The gastrointestinal (GI) tract of dairy cattle is colonized by a wide variety of microorganisms, which coexist in a symbiotic relationship with the animal. This resident microbiota is central to the host productive and health responses [1]. As efforts continue to explore host-microbiome interactions towards increasing cow productivity [2], identification of factors that influence the composition of the GI microbiota is warranted. Delivery of fresh feed is a potent stimulus that can alter dairy cattle behavioral patterns. Previous studies have associated increased feeding frequency with greater feeding time, more evenly distributed meals throughout the day, and higher dry matter intake (DMI) [3,4]. Furthermore, changes in feeding frequency can modify nutrient digestibility and fermentation profiles in the rumen and lower GI tract [5,6]. In turn, diurnal fluctuations in the GI environment can impact the microbial community composition [7]. For instance, Lengowski et al. [8] found the abundance of rumen microbial groups to be correlated with fermentation variables such as specific volatile fatty acids (VFA), acetate: propionate ratio, pH, and ammonia (NH_3_). Delivery of feed 1× or 2×/d is a standard feeding practice in dairy operations [9]; however, evaluation of the diurnal dynamics of the GI microbiota under these feeding frequencies is limited.

The rumen microbiota is crucial for feed digestion and performance in dairy cattle [10,11]. However, the microbiota of other sections of the GI tract also have considerable contributions to the animal nutrition and well-being. In the large intestine, microbial fermentation of plant structural polysaccharides yields energy in the form of VFA [12] and research suggests that the bacterial community from this GI section is associated with feed efficiency phenotype [13]. Characterization of the bacteriome across the GI tract has revealed different community profiles between the rumen and lower gut [14]. Moreover, breed has also been identified as a factor that differentiates the bacterial community structure across the GI tract [15,16]. Therefore, diurnal patterns from the bacterial community could potentially differ between GI sections or breeds.

The objective of this study was to evaluate the effect of feeding frequency on behavioral patterns and diurnal fermentation and bacteriome profiles of the rumen and feces from two main dairy breeds, Holstein and Jersey. We hypothesized that increasing feeding frequency will influence the rumen and lower gut environments through changes in feeding behavior and we further hypothesized that these environmental variations would promote changes in the diurnal profiles of their respective resident bacteriome.

## Material and methods

### Animals, housing, and diet

All management and experimental procedures involving animals were approved by the Mississippi State University Institutional Animal Care and Use Committee (IACUC #18–313).

This study was conducted in November 2019 and included 10 lactating Holstein cows with an initial 603 ± 71 (mean ± SD) kg of body weight, 1.5 ± 0.70 parities, 254 ± 8.5 days in milk, and 24.4 ± 3.85 kg/d of milk yield and 10 Jersey cows with an initial 415 ± 54 kg of body weight, 1.7 ± 1.49 parities, 252 ± 13.7 days in milk, and 16.8 ± 3.77 kg/d of milk yield. All animals were housed in a free-stall barn within the same pen at the Bearden Dairy Research Center (Starkville, MS). The experimental pen was equipped with 22 deep-bedded sand stalls for a stocking density of 91%. Cows were milked 2×/d (at 0300 and 1500 h) in a double eight parallel milking parlor at which animals were moved as a group. All animals were under the same management and total mixed ration (TMR) and had free access to water troughs. The diet was balanced to meet or exceed the animal’s requirements [17] in a 53:47 forage-to-concentrate ratio (dry matter (DM) basis; S1 Table).

### Experimental design and feeding protocol

At the beginning of the experiment, Holstein and Jersey cows were randomly allocated to one of the following feeding frequencies: (1) TMR delivered 1×/d (at 0600 h) or (2) TMR delivered 2×/d (at 0600 and 1800 h) in a 2 × 2 factorial arrangement of treatments. Within breed, initial body weight (*P* = 0.18), days in milk (*P* = 0.44), and milk yield (*P* = 0.87) were similar between treatments. Cows were fed individually using the Calan Broadbent Feeding System (American Calan Inc., Northwood NH) and went through a 14-d adaptation period. At the end of the adaptation period, cows were able to access their assigned feed bin without assistance and had a steady individual intake (mean variation ≤ 10%). The adaptation period was followed by a 14-d collection period. During each feeding, orts were collected and weighed before the fresh TMR was mixed and offered to the cows. The amount of TMR offered at each feeding to each cow was adjusted based on the individual orts data from the previous day for *ad libitum* consumption targeting orts between 5 and 10% (as is basis) of the total TMR offered. No signs of clinical mastitis were observed during milking or laminitis during daily management and all cows completed the study. After completion of the study, all cows returned to the farm management protocols.

### Measurements and sampling

#### Intake measurement and milk yield

On d 20, 21, 27 and 28, TMR and refusals were collected on every feeding and immediately frozen at -20°C until further analysis. At the end of the study, TMR and refusals were composited to determine DM (48 h at 55°C in a forced-air oven). A subsample from the TMR composite was analyzed for chemical composition (Standard Package; Cumberland Valley Analytical Services Inc., Hagerstown, MD). Daily individual intake from each cow was later calculated as the difference between the amount of feed offered and refused (DM basis). Milk yield for each animal was recorded daily during the entire experiment (Westfalia Surge Metatron 12 Milk Meter; GEA Farm Technologies, Oelde, Germany).

#### Behavioral observations

On d 23 and 24, cows were monitored continuously for 48 h to record direct behavioral observations using scan sampling at 15 min intervals by four trained observers. For each observation period, an observer rounded the experimental pen from the outside (to prevent disturbance), identified each animal via neck identity collar or ear tag, and recorded each animal’s activity. The following activities were used to describe the cows daily time budget: lying (at the stall in a resting position), eating (animal at the assigned feeding gate with the Calan door unlocked and head in the feed bin), drinking (animal directly in front of the water trough and actively drinking or immediately after), standing/walking at the isle or stall, and milking (from the moment the first animal was taken to the parlor until the last animal returned to the experimental pen). Cows were assumed to perform the recorded activity during the complete 15 min interval until the next observation. The sum of all the time budget activities equals 24-h. Simultaneously and independently from the aforementioned activities, rumination activity (recognized as continuous jaw movements due to remastication of feed or with signs of regurgitation) was recorded and classified according to the animal’s posture (lying or standing) and the number of visits to the feeding gate (feeding event from the moment the cow unlocked the Calan door and interacted with the feed bin to its exit) was also recorded. For every visit to the feeding gate, the time of entrance and exit from the feed bin were registered. During the observation period, no management activities out of the routine were performed.

#### Rumen and fecal sampling

On d 27, a total of 5 concomitant collections of rumen and fecal samples were performed at 6 h intervals (0 [pre-feeding am], 6, 12 [pre-feeding pm], 18, and 24 [pre-feeding am] h). Rumen samples were collected using a gastroesophageal apparatus that consisted of a reinforced vinyl tube coupled on one end to a metal strainer and on the other end to a 500 mL sterile collection flask that was also connected to a vacuum pump (model 1HAB25BM100X, Gast, Benton Harbor, MI). Ruminal contents were collected as described by Paz et al. [15]. Briefly, a Frick speculum was used to pass the tube through the oral cavity into the rumen. The pump was turned on only after the tube was located in the rumen with the first 200 mL of rumen sample discarded to prevent saliva contamination and the subsequent 200 mL of rumen sample collected. To prepare the sample for bacterial community analysis from each animal, particles attached to the strainer were removed and then mixed with 40 mL of rumen fluid to represent the solid and liquid fractions. After each sample, the tubing, metal strainer, and Frick speculum were washed with warm water and a new sterile collection flask was placed to avoid sample carryover. In addition, three subsamples (40 mL each) of rumen fluid were poured into polypropylene conical tubes for pH, VFA, and NH_3_ analyses. Approximately 200 g of stool were collected from each cow via fecal grab and stored in inert, polyethylene cups. Fecal samples were used as a proxy for the lower gut bacterial community [18]. Immediately after sampling, rumen and fecal pH were measured using a portable pH meter (handheld pH meter, Oakton, Vernon Hills, IL) by direct insertion. All remaining samples were immediately placed on ice for less than 10 min before being moved to a -20°C freezer on-farm. Once the collection of all samples was completed, samples were transferred to the laboratory and stored at -80°C until analysis.

#### VFA and NH_3_ analyses

Before analysis, VFA and NH_3_ samples were thawed and filtered through four layers of cheesecloth. NH_3_ concentration was determined using a portable meter (Orion meter model 290, Thermo Scientific Inc., Waltham, MA) coupled to an NH_3_ ion selective electrode (Orion 9512 ammonia sensing electrode Thermo Scientific Inc., Waltham, MA) [19]. For VFA analysis, filtered samples were acidified with 25% metaphosphoric acid (w/v), centrifuged at 18,000 × g for 20 min, and the resulting supernatant was analyzed using a 7890A gas chromatography system equipped with a DB-FATWAX Ultra Inert column (30 m × 0.25 mm, 0.25 μm), a 5975C inert XL MSD with triple-axis mass detector, a 7693 series autosampler (Agilent Technologies, Santa Clara, CA, USA). Ionization was performed in an electron impact mode at 70 eV and a selected ion monitoring (SIM) mode was used to acquire ion abundance. Volatile fatty acids were quantified by an internal standard calibration with authentic volatile fatty acid standards. Isobutyric acid and isovaleric acid averaged 0.6 and 0.8 mol/100 mol across samples and were not included in further analyses.

#### DNA extraction and amplification

Genomic DNA from rumen and fecal samples was extracted using the Mag-Bind Stool DNA Kit (Omega Bio-Tek, Norcross, GA, U.S.), according to the manufacturer’s protocol with a minor modification. For the lysing step, the protocol was adapted to use a mixer mill (Retsch MM 200) set up for 10 min at 25 Hz. The quality of the resulting DNA was checked using gel electrophoresis (1% agarose gel) and the concentration was measured with a NanoDrop One spectrophotometer (Thermo Fisher Scientific, Wilmington, DE, USA). Bacterial amplicon libraries of the V4 region from the 16S rRNA gene were prepared as described by Paz et al. [20]. The resulting amplicons were normalized to a concentration of 1–2 ng/μl using the SequalPrep^TM^ normalization plate. Pooled libraries were sequenced using the Illumina MiSeq platform (Illumina, San Diego, CA, USA). Raw sequences are available at the NCBI Sequence Read Archive (SRA) under the accession no. SRP271418.

#### Bioinformatics analysis

Raw sequences were analyzed using the QIIME 2 package [21]. Analysis included denoising of raw sequences using Deblur [22], clustering of quality-filtered reads to amplicon sequence variants (ASV), and rarefaction to an even depth (4,600 reads). Sampling depth was assessed with rarefaction curves and the Good’s coverage index [23]. Visualization of taxonomic data was done with heat trees using the "metacoder" package [24] from the R software (v3.6.1) [25]. Alpha diversity was calculated using the observed ASV and Shannon diversity indices and beta diversity was calculated using the weighted UniFrac distances via the q2-diversity plugin. Detailed description of the bioinformatic pipeline used in this study is available at: https://github.com/pazlabgit/feeding_freq_2021.

### Statistical analyses

Prior to analyses, dry matter intake, milk yield, and behavioral data were tested for normality using the Shapiro-Wilk test and screened for outliers (1.5 times the interquartile range above the third quartile or below the first quartile) with no observations removed. Data were then analyzed using the MIXED procedure of SAS 9.4 (SAS Institute Inc., Cary, NC). Analyses for DMI and milk yield were performed on experimental period means (14 d) and for behavioral observations on collection period means (2 d). Models included the fixed effects of feeding frequency, breed, and their interaction. Rumen and fecal pH and rumen VFA data were analyzed as repeated measures and the compound symmetry, autoregressive, heterogeneous compound symmetry, and unstructured covariance structures were tested based on the lowest Akaike’s information criteria.

Statistical analyses for alpha and beta diversity metrics were performed in R v 3.5.1 [25]. Alpha diversity indices were compared using the Kruskal-Wallis test and post-hoc pairwise comparisons were done using the Wilcoxon rank-sum test. Beta diversity was analyzed through a permutational analysis of variance (PERMANOVA) using the weighted UniFrac distance matrix as input (adonis function from the "vegan" package [Oksanen et al. [26]]) and was visualized using the principal coordinate analysis (PCoA) plot. The linear discriminant analysis (LDA) effect size (LEfSe) method [27] was used to identify differentially abundant features across collection times. The *P*-values were adjusted for multiple testing using the false discovery rate (FDR) method [28]. For production, behavioral, and fermentation variables, statistical significance was declared at *P* ≤ 0.05 and tendencies were discussed at 0.05 < *P* ≤ 0.10. For bacterial community metrics, statistical significance was declared at *P* ≤ 0.05.

## Results and discussion

### Production responses

There were no interactions between feeding frequency and breed on production responses (*P* ≥ 0.15; Table 1). No differences were observed in DMI when feeding 1 or 2×/d. According to Hart et al. [4], feeding frequency increased DMI only after cows were fed 3×/d with no difference observed between 1 or 2×/d feedings. In contrast, a survey of freestall herds showed an increase in DMI of 1.42 kg when feeding frequency was increased from 1 to 2×/d [29]. In the current study, increasing feeding frequency tended (*P* = 0.07) to increase milk yield which agrees with previous reports [29,30], but feed efficiency, expressed as kg of milk/kg of DMI, did not differ (*P* = 0.15). For breed responses, Holstein cows had a higher DMI (*P* = 0.07) than Jersey cows; however, the opposite was observed for DMI capacity (DMI/BW; *P* = 0.05). A similar response was reported by Beecher et al. [31] and the authors attributed this to a heavier reticulorumen, as a proportion of BW, in Jersey compared to Holstein cows. The capacity of the reticulorumen is a main animal factor regulating DMI [32], thus a proportionally heavier reticulorumen suggests that the physical fill effect on DMI capacity is lower on Jersey than Holstein cows. Milk production was higher (*P* < 0.01) in Holstein cows compared to Jersey cows (*P* < 0.01), but feed efficiency was similar between breeds and averaged 1.29 kg of milk/kg of DMI (*P* = 0.19). In a study that included data from 13 dairy herds, feed efficiency ranged from 1.11 to 1.67 kg of milk/kg of DMI [33].

**Table 1:** Effects of feeding frequency and breed on production responses and behavioral observations.

	Feeding frequency							
	1×/d		2×/d			*P*-value^1^		
Measure^2^	Holstein	Jersey	Holstein	Jersey	SEM	FF	B	FF×B
DMI, kg/d	17.6	17.0	19.3	14.1	1.48	0.70	0.07	0.15
DMI capacity^3^, kg/100 kg of BW	3.10	4.34	3.06	3.30	0.36	0.15	0.05	0.18
Milk yield, kg/d	23.8	16.2	26.7	17.7	1.16	0.07	< 0.01	0.55
Feed efficiency^4^, kg/kg	1.38	0.95	1.44	1.39	0.42	0.18	0.19	0.31
Lying, h/d	15.0	13.1	15.7	13.8	0.83	0.39	0.04	0.98
Standing, h/d								
Eating	3.77	4.17	3.20	4.03	0.38	0.35	0.13	0.58
Drinking	0.15	0.43	0.23	0.23	0.08	0.46	0.12	0.12
In alley or stall	3.73	4.98	3.53	4.58	0.73	0.68	0.13	0.89
Total	7.65	9.57	6.95	8.82	0.83	0.40	0.04	0.98
Rumination, min/d								
Lying	358	268	378	303	20.7	0.21	< 0.01	0.72
Standing	43.5	54.0	22.5	36.0	12.9	0.15	0.37	0.91
Total	402	322	400	339	17.5	0.67	< 0.01	0.61
Visits to the feeder, n/d	10	13	10	13	1.46	0.95	0.07	0.74

^1^FF = feeding frequency; B = breed.

^2^Times for direct behavioral observations were recorded under the assumption that the registered activity was performed during the complete 15 min interval. Lying + Total Standing + 1.3 h Milking = 24 h.

^3^DMI capacity = DMI/BW.

^4^Feed efficiency = MY/DMI.

### Behavioral responses

Similar to production responses, there were no interactions between feeding frequency and breed on behavioral observations (*P* ≥ 0.12; Table 1). The average time budget for the animals was distributed as follows: 60% lying, 18% standing in the alley or stall, 16% eating, 5% milking, and 1% drinking. No significant differences were observed for time spent standing in the alley or stall, eating, or drinking between feeding frequencies (*P* ≥ 0.35) or breeds (*P* ≥ 0.12). Eating and drinking diurnal patterns matched mean responses and did not differ (*P* ≥ 0.34) by feeding frequency in both breeds (S1 Fig). These results are consistent with others observing no differences in diurnal DMI [4] and water consumption [34] patterns in lactating cows fed 1 and 2×/d. Lying time was significantly higher (*P* = 0.04) for Holstein when compared to Jersey cows. Similarly, Munksgaard et al. [35] reported that Holstein cows spent more time lying while Jerseys cows took a greater number of steps throughout the day. In the current study, Jersey cows tended (*P* = 0.07) to visit more times their assigned feeding gates. Furthermore, a positive correlation between rumination time and lying time was found in dry cows [36]. In this study, differences between breeds support this correlation since both rumination time and lying time were greater in Holstein compared to Jersey cows. Overall, cows spent 89% and 11% of their rumination time lying and standing, respectively. A factor that could influence the rumination behavior that was not measure in this study is sorting. Cows normally sort against long forage particles [37] and as the TMR particle size increases, behavioral responses such as ruminating and eating time have been observed to linearly increase [38]. In group-fed cows, DeVries et al. [3] reported a curvilinear increase throughout the day in the neutral detergent fiber content of the diet which reflected sorting in feeding frequencies from 1×/d up to 4×/d, while in tie stall cows, Macmillan et al. [39] observed no differences in sorting between feeding frequencies of 1 and 3×/d. This discrepancy underlines the need to further elucidate the relationships between feeding frequency, sorting, and behavioral parameters. It is important to note that observations during 2 d with 15 min intervals adequately describe lying and standing behavior, but for eating and rumination shorter min intervals than the one used in this study are recommended [40,41]. Yet, observed rumination and eating times are in agreement with previous works [42,43].

### Fermentation characteristics

Effects of feeding frequency, breed, and time on rumen and fecal pH and rumen NH_3_, total VFA concentration, and molar proportions of individual VFA are shown in Table 2. Interactions between feeding frequency and breed for the rumen and fecal fermentation variables were not significant (*P* ≥ 0.33). Rumen and fecal pH varied across collection times (*P* < 0.01), ranging from 6.50 to 6.75 at 6 and 24 h and from 6.63 to 7.02 for collection times at 24 and 12 h, respectively (S2 Table). Holstein cows had a lower (*P* = 0.01) rumen pH compared to Jersey cows. On average, Holstein cows consumed 0.9 kg of concentrate more than Jersey cows. Consumption of grain-rich concentrates increases the production of VFA which can promptly dissociate decreasing the rumen pH [44]. Total VFA concentration was higher (*P* = 0.02) in Holstein compared to Jersey cows. Holstein cows ruminated more than Jersey cows potentially increasing saliva production. In dairy cattle, buffers in saliva, bicarbonate and hydrogen phosphate, can aid to remove around 37% of total hydrogen from to rumen to maintain physiological pH [45]. For Holstein cows in this study, it can only be speculated that the salivary buffer was not enough to compensate pH to that observed in Jersey cows which consumed less concentrate. Nevertheless, a healthy rumen environment was maintained as reflected by pH values ≥ 6.5 across times [46]. Similar to rumen pH, fecal pH differed by breed (*P* = 0.07). Following a grain challenge, Luan et al. [47] observed the patterns of fecal and rumen pH to be similar, but the fecal pH lagged in time. In the current study, a lower fecal pH in Holstein cows compared to Jersey cows suggests that the higher DMI resulted in more fermentable carbohydrates reaching the hindgut. For rumen NH_3_, a significant feeding frequency × time interaction was observed where NH_3_ concentration was lower at collection time 0 h (7.19 vs 9.21 mg/dL) and higher at collection time 24 h (10.2 vs 6.78 mg/dL) in 1×/d compared to 2×/d feeding. No effect of feeding frequency was detected for total VFA concentration or the molar proportion of individual VFA (*P* ≥ 0.12). Similar diurnal feed intake patterns between feeding frequencies support these observations. In addition, similar responses have been reported when increasing feeding frequency from 1 to 4×/d in Holstein heifers [48]. However, total VFA concentration was higher during the first two collection times compared to the remaining times (*P* < 0.01; S2 Table). Difference between collection times 0 and 24 h, which represented pre-feeding am times, could be related to a day effect on total VFA concentration [49].

**Table 2:** Effects of feeding frequency, breed, and time on rumen and fecal pH and rumen fermentation parameters.

	Feeding frequency							
	1×/d		2×/d			*P*-value^1^		
Measure	Holstein	Jersey	Holstein	Jersey	SEM	FF	B	T
Rumen pH	6.48	6.81	6.56	6.72	0.08	0.97	0.01	<0.01
Feces pH	6.71	6.80	6.67	6.85	0.07	0.91	0.07	<0.01
Ammonia, mg/dL	8.54	9.78	7.97	8.82	0.85	0.40	0.26	0.50
Total VFA, mM	198	157	181	158	12.4	0.53	0.02	<0.01
VFA, mol/100 mol								
Acetic acid	70.4	71.2	69.8	70.6	1.09	0.60	0.49	0.12
Propionic acid	16.8	16.2	18.0	16.0	1.01	0.67	0.26	0.32
Butyric acid	11.4	11.7	11.4	11.8	0.58	0.93	0.55	0.23
Valeric acid	1.03	1.10	1.12	1.22	0.08	0.23	0.33	0.14

^1^FF = feeding frequency; B = breed; T = time (0 [pre-feeding am], 6, 12 [pre-feeding pm], 18, 24 [pre-feeding am] h). All interactions were not significant (*P* ≥ 0.15) but the FF×T interaction for ammonia (*P* = 0.01).

### Sequencing information

A total of 5,971 ASV were identified across the rarefied samples. Samples with less than 4,600 quality-filtered reads were removed from the analyses. Rarefaction curves showed a similar coverage for feeding frequency (1 and 2×/d feed deliveries), breed (Holstein and Jersey), sample type (rumen and feces), and collection time (0 [pre-feeding am], 6, 12 [pre-feeding pm], 18, and 24 [pre-feeding am] h) (S2 Fig). Based on the Good’s coverage index, the sequencing depth in this study enable the characterization of at least 95% of the bacterial community across samples.

### Sample type

**Fig 1:**
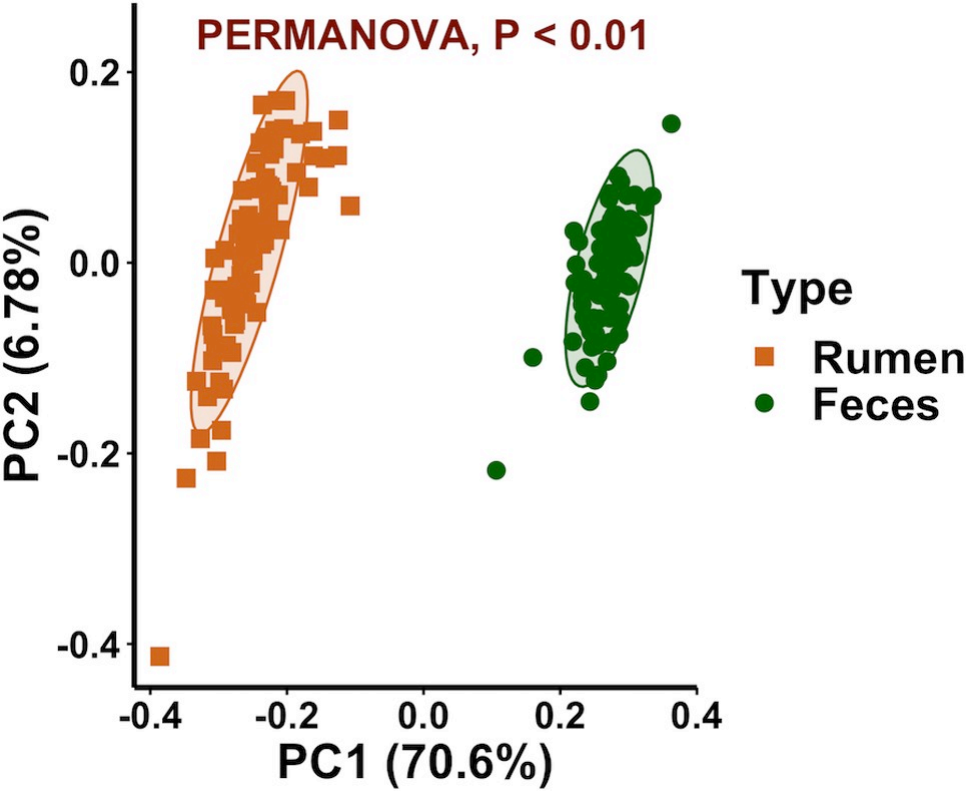
Principal coordinate analysis displaying clustering of bacterial communities by sample type. Beta diversity calculated by using the weighted UniFrac distances. Ellipses represent the 95% confidence intervals.

**Fig 2:**
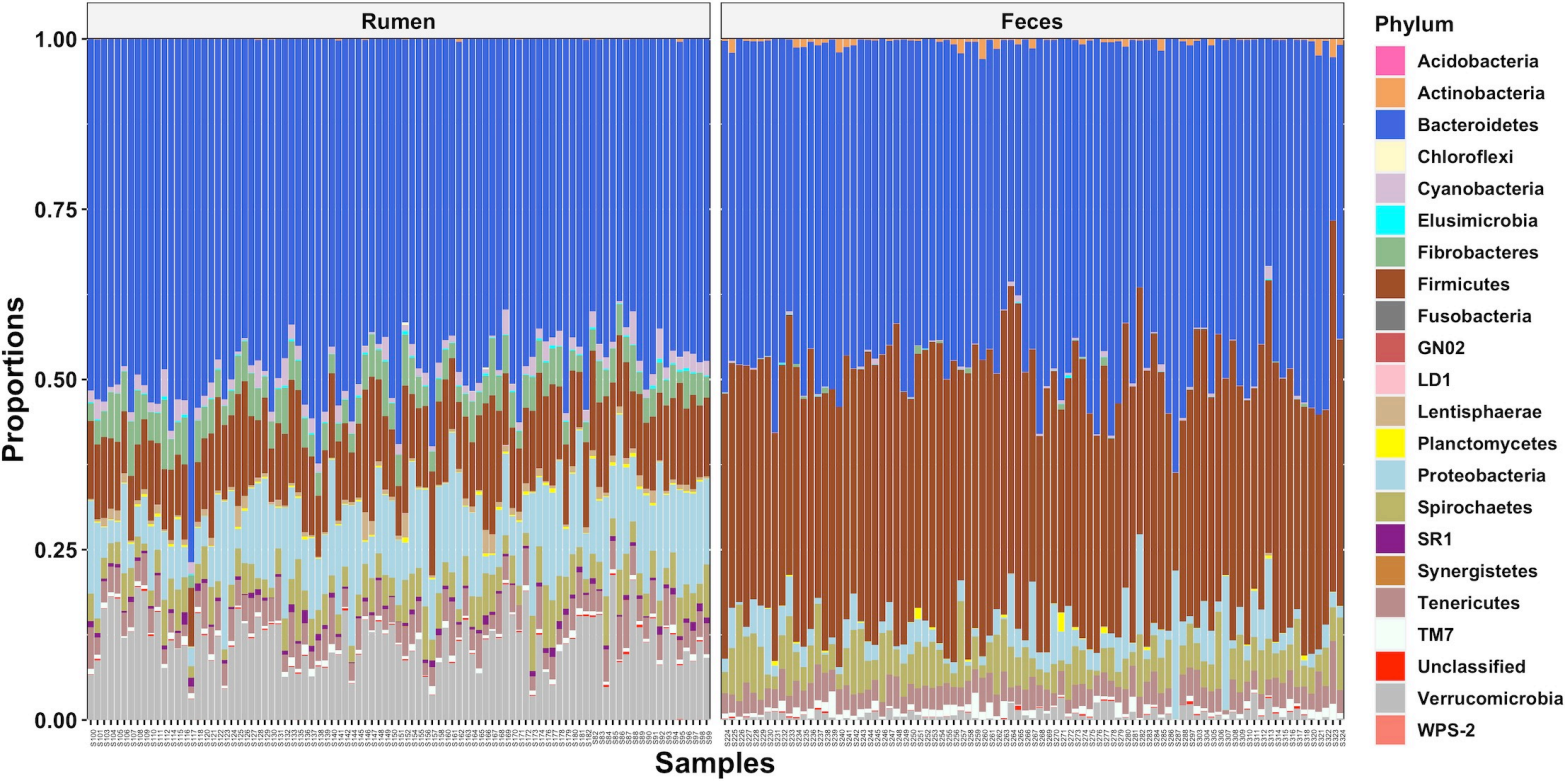
Phyla level classification of the bacterial communities from the rumen and feces of dairy cows.

A significant sample type effect on the bacterial community composition was observed (*P* < 0.01). This was clearly visualized using a PCoA plot which revealed rumen and fecal samples clustered separately (Fig 1). The taxonomy analysis showed that both rumen and fecal bacterial communities were dominated by members of the *Bacteroidetes* and *Firmicutes* phyla (Fig 2) but in different proportions, 49.0 and 11.8% of the total reads in the rumen samples and 47.6 and 37.3% of the total reads in the fecal samples. Other abundant phyla in the rumen samples were *Verrucomicrobia*, *Proteobacteria*, *Fibrobacteres*, *Spirochaetes*, *Tenericutes*, and *Cyanobacteria* (11.6, 10.9, 4.5, 4.2, 3.8, and 1.7% of the total reads) and in the fecal samples were *Spirochaetes*, *Proteobacteria*, *Tenericutes*, *TM7*, and *Verrucomicrobia* (5.2, 3.7, 3.4, 0.9, and 0.9% of the total reads). Heat trees displaying the classification for all the taxonomic levels in the rumen and fecal bacterial communities are shown in S3 Fig. Considering previous studies that characterized the bacteriome across the GI tract of Nelore and Holstein cattle also observed site specificity [14,18], further bacteriome analyses in this study were performed within sample type (rumen or feces).

### Alpha diversity

**Fig 3:**
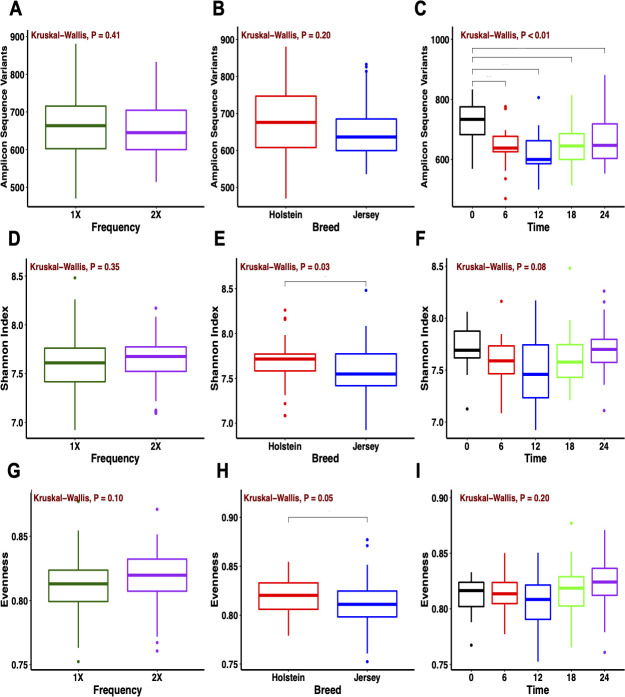
Alpha diversity metrics of the rumen bacterial community. (A, B, C) Observed amplicon sequence variants, (D, E, F) Shannon index, and (G, H, I) evenness for feeding frequency, breed, and collection time (0 [pre-feeding am], 6, 12 [pre-feeding pm], 18, and 24 [pre-feeding am] h).

Alpha diversity was evaluated using the observed ASV for richness, the Shannon index for diversity, and the Pielou’s index for evenness. In the rumen, species richness did not differ by feeding frequency (*P* = 0.41) or breed (*P* = 0.20), whereas species richness was higher (*P* < 0.01) at collection time 0 compared to the remaining times (Fig 3). Species diversity was similar between feeding frequencies (*P* = 0.35) and collection times (*P* = 0.08) and differed by breed (*P* = 0.03). A higher rumen methanogen diversity has been reported in Holstein compared to Jersey cows [50]. Yet, studies have reported similar alpha metrics associated with the rumen bacterial community between the Holstein and Jersey breeds under the same diet [15,51]. Evenness was similar for feeding frequency and collection time (*P* ≥ 0.10), but Holstein cows exhibited higher evenness compared to Jersey cows (*P* = 0.05). In fecal samples, bacterial richness was similar for feeding frequency, breed, and collection time (*P* ≥ 0.32; S2 Fig). Similar to the rumen, fecal samples from Holstein cows exhibited higher diversity and evenness (*P* ≤ 0.04) than Jersey cows. Overall, the bacterial community diversity from both the rumen and feces was affected by breed but not by feeding frequency. In addition, the bacterial community richness from the rumen was affected by collection time.

### Beta diversity

#### Feeding frequency and breed effects

**Fig 4:**
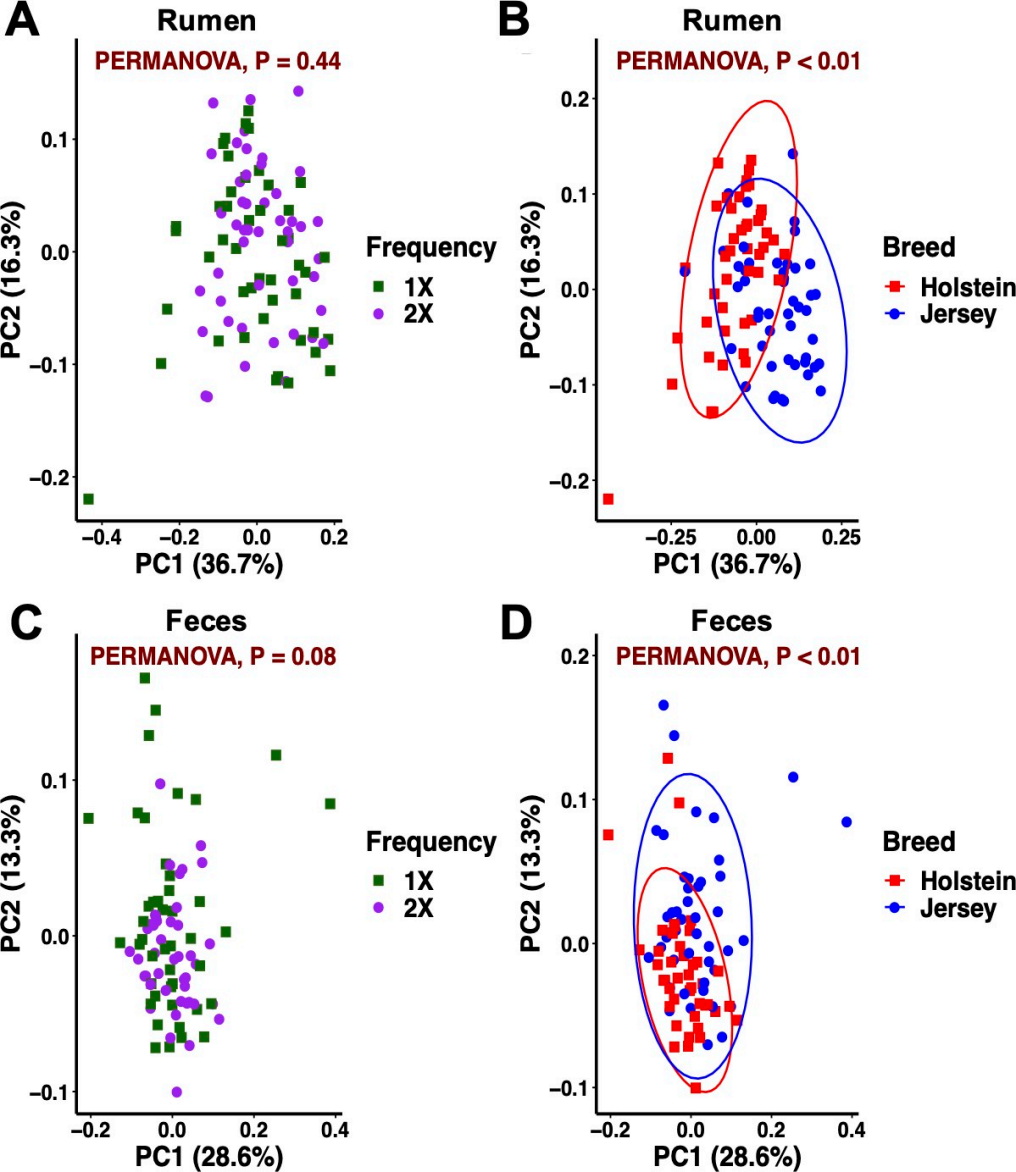
Principal coordinate analysis of the bacterial community for feeding frequency and breed within sample type in dairy cows. (A, B) Rumen and (C, D) feces. Beta diversity calculated by using the weighted UniFrac distances. Ellipses represent the 95% confidence intervals.

Weighted UniFrac distances did not separate the rumen bacteriome community by feeding frequency but by breed (Fig 4A and 4B). Breed differences in the composition and function of the rumen microbiome have been reported [15,52]. In addition, a potential role of the host genetics shaping the rumen microbiota is starting to emerge [53,54]. Contrarily to our hypothesis, the rumen bacteriome structure was not influenced by feeding frequency (*P* = 0.44). Feeding frequency can influence the flow of substrates and fermentation patterns in the rumen through changes in feeding behavior such as total feeding time, distribution of feeding time throughout the day, meal size, and total DMI [4,39]. In turn, a dynamic rumen environment can influence the bacteriome profile. In the current study, we observed parallel diurnal feed intake patterns between feeding frequencies in both breeds. This promoted similar DMI responses and similar fermentation profiles with no overall differences in pH, NH_3_ and total VFA concentrations, and molar proportion of individual VFA. In turn, under comparable rumen environments, the bacteriome composition did not differ by feeding frequency. Following ruminal observations, the fecal bacterial community composition was differentiated by breed but not by feeding frequency (Fig 4C and 4D). In agreement with our results, Fan et al. [16] reported significant differences in the fecal microbial population of preweaned calves from 6 breeds of beef cattle. Similar fecal pH by feeding frequency suggests a similar environment in the lower gut and supports no effect on the bacteriome composition. These results show that both site and breed are factors that drive the bacterial community composition across the gastrointestinal tract.

#### Diurnal profiles

**Fig 5:**
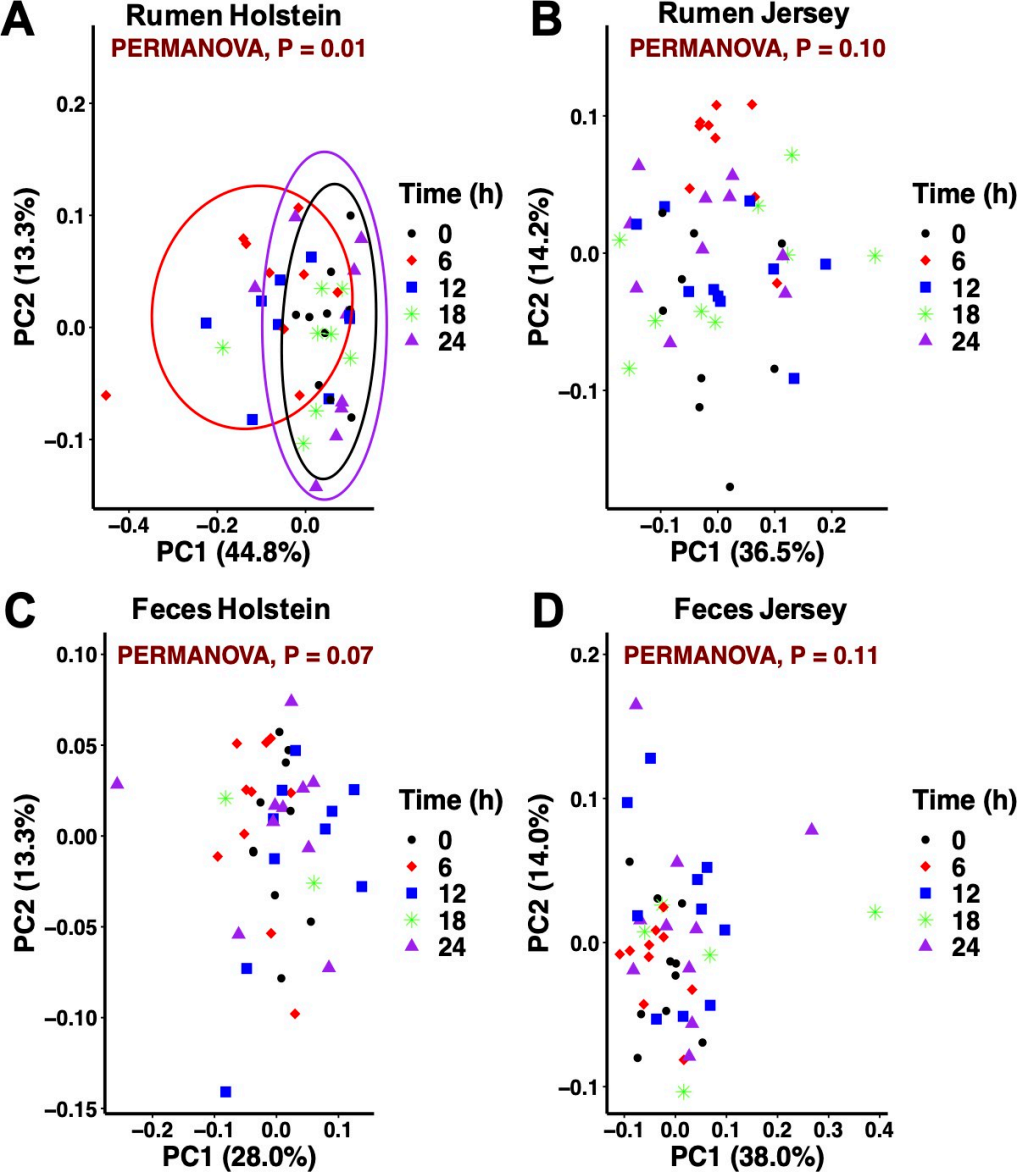
Principal coordinate analysis of the bacterial community across times for each sample type within breed in dairy cows. (A, B) Rumen and (C, D) feces. Collection time = 0 (pre-feeding am), 6, 12 (pre-feeding pm), 18, and 24 (pre-feeding am) h. Ellipses represent the 95% confidence intervals. (A) Beta diversity at 6 h differed from that at 0 and 24 h in the rumen bacterial community from Holstein cows.

Diurnal oscillations of the bacteriome composition in the GI tract could result in functional profiles that influence the host metabolic balance [55]. However, characterization of the diurnal patterns from the GI bacteriome composition in the main dairy breeds and identification of factors that potentially influence these patterns is scarce. The current study characterized the diurnal profile of the microbial community in the rumen and feces over 5 collection times equally distributed along a 24 h period. Since feeding frequency did not influence bacteriome composition, diurnal patterns were assessed by breed and sample type. Diurnal differences in the composition of the rumen bacteriome were seen in Holstein but not in Jersey cows (Fig 5). Specifically, bacteriome composition at 6 h differed from that at 0 and 24 h. The pH value at 6 h was the lowest and differed (*P* < 0.01) from the other two times (6.50, 6.63, and 6.75, respectively; S2 Table). In a study with Holstein cows fed diets with a 30:70 forage-to-concentrate ratio, Shaani et al. [7] also observed changes in the rumen bacteriome linked to pH as the community 10 h after feeding differed from the communities 1 h prior to feeding and 4 h after feeding where pH across those times averaged 6.2, 7.1, and 6.8, respectively. Welkie et al. [56] suggested that the diurnal variation in composition of the liquid-associated bacteria is greater than that of the solid-associated bacteria. The latter should be viewed with caution since that study only used two Holstein cows and characterization of the bacteriome was limited to 155 amplicon lengths using automated ribosomal intergenic spacer analysis. The rumen sample collection method used in the present study, which included esophageal tubing with the addition of particles retained in the strainer, is adequate to describe the rumen bacterial community [15]. Similar rumen bacteriome composition across times for Jersey cows could be related to higher pH values compared to Holstein cows. For fecal samples, no differences in the bacterial community composition were observed across collection times in both breeds (*P* ≥ 0.07) despite changes in pH. To further explore diurnal variations at the ASV level, LEfSe was used to identify differentially abundant features in both the rumen and fecal samples (S3 Fig). Including data from both breeds, the relative abundance of 195 ASV differed at least once during the collection times in the rumen, whereas 302 ASV differed at least once during the collection times in the feces. The majority of abundant (> 1% relative abundance) ASV that significantly varied during the day in the rumen belonged to the *Prevotella* or unclassified genera, whereas in the feces ASV also belonged to the *5-7N15* genus. Variation of specific ASV throughout the day could reflect fermentation dynamics of the different dietary components in both the rumen and lower gut. Dairy farms will continue to grow in size and rely more on technology [57]. The use of automatic feeding systems allows more frequent delivery of fresh feed compared to conventional industry practices. Increasing feeding frequency above 2×/d can influence both the rumen and postrumen conditions [39,58]; however, how these changes impact the relationships between the gastrointestinal environment, microbiome structure and function, and production and health responses are areas that require further research efforts.

## Conclusions

The bacterial community profile of the GI tract in dairy cows can be altered by multiple factors. This study evaluated feeding frequency as a potential factor that could impact the GI bacterial community through changes in feeding behavior. Feeding frequency of 1 or 2×/d resulted in similar feeding patterns and fermentation profiles and did not differentiate the bacterial community in the rumen or feces in both Holstein and Jersey cows. However, breed and sample type significantly influenced the bacteriome composition. No major diurnal fluctuations were observed in the fecal bacterial community from both breeds but were identified in the rumen bacterial community from Holstein cows which appeared to follow pH responses.

## Supporting information

S1 Fig Eating and drinking diurnal patterns from Holstein and Jersey cows. Vertical dashed lines represent feeding times.
(TIF)

S2 Fig Rarefaction curves of the bacterial community from dairy cows. (A) Feeding frequency, (B) breed, (C) sample type, and (D) collection time (0 [pre-feeding am], 6, 12 [pre-feeding pm], 18, and 24 [pre-feeding am] h). Samples were rarefied at an even depth of 4,600 reads and values represent medians from 10 iterations.
(TIF)

S3 Fig Heat trees displaying the taxonomic profiles of the bacterial communities from the rumen and feces of dairy cows. Color and size of the nodes (circles) and edges (lines) correspond to the relative abundance of the respective taxonomic rank.
(TIF)

S4 Fig Alpha diversity metrics of the fecal bacterial community. (A, B, C) Observed amplicon sequence variants, (D, E, F) Shannon index, and (G, H, I) evenness for feeding frequency, breed, and collection time (0 [pre-feeding am], 6, 12 [pre-feeding pm], 18, and 24 [pre-feeding am] h).
(TIF)

S5 Fig Diurnal profile of the differentially abundant amplicon sequence variants shown at the genus rank. (A) Rumen and (B) fecal samples from both Holstein and Jersey cows. Amplicon sequence variants with a relative abundance > 1% are presented. Collection time = 0 (pre-feeding am), 6, 12 (pre-feeding pm), 18, and 24 (pre-feeding am) h.
(TIF)

S1 Table Ingredient and chemical composition of the diet. (DOCX)

S2 Table Diurnal patterns of the rumen and fecal pH and rumen fermentation parameters. (DOCX)
