# Supplementary figures and images for: Influence of industry standard feeding frequencies on behavioral patterns and rumen and fecal bacterial communities in Holstein and Jersey cows
Source: PLoS One. 2021 Mar 5;16(3):e0248147. doi: 10.1371/journal.pone.0248147 (PMC7935240; doi:10.1371/journal.pone.0248147)

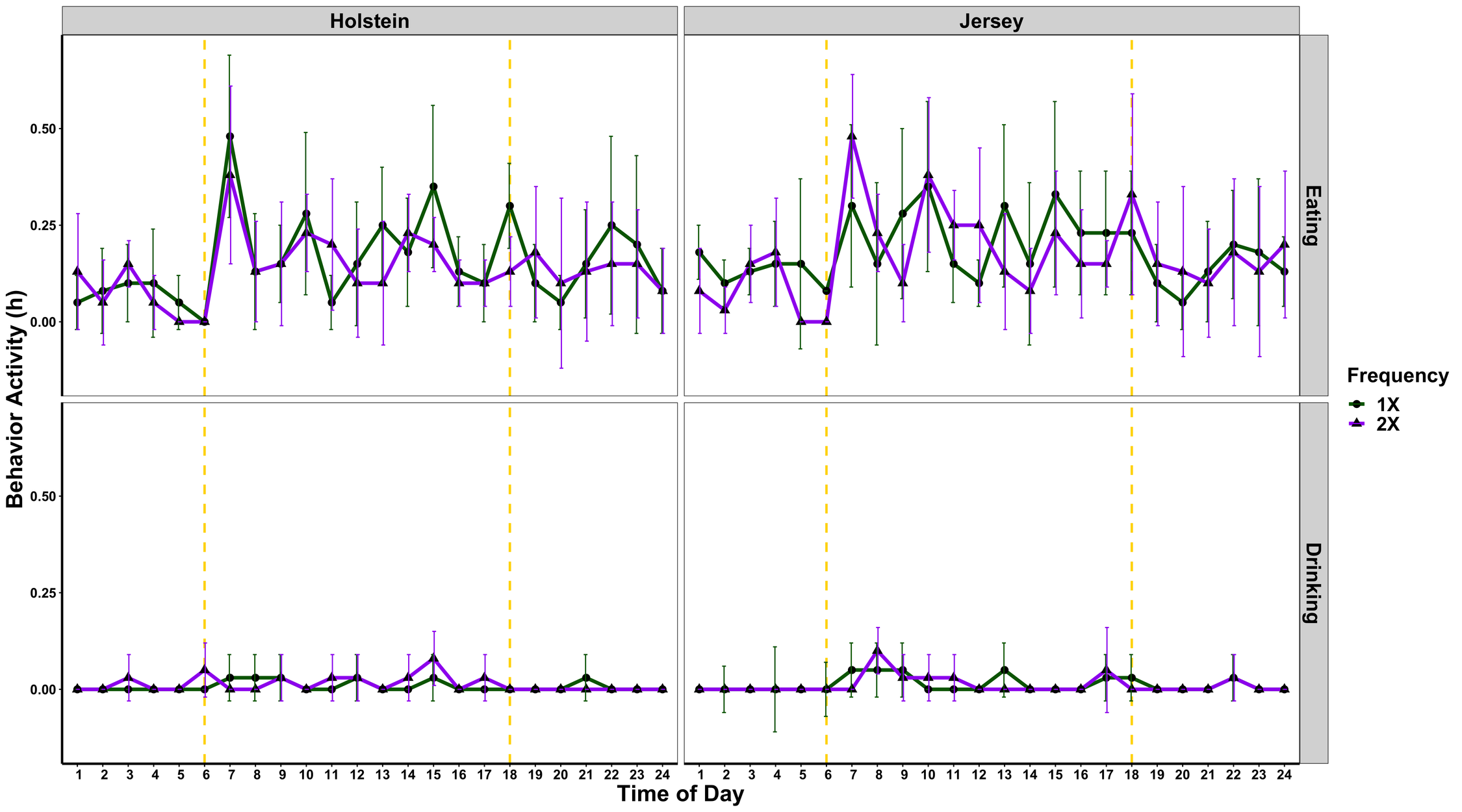

Supplement: S1 Fig — Vertical dashed lines represent feeding times. (TIF) [file pone.0248147.s001.tif]

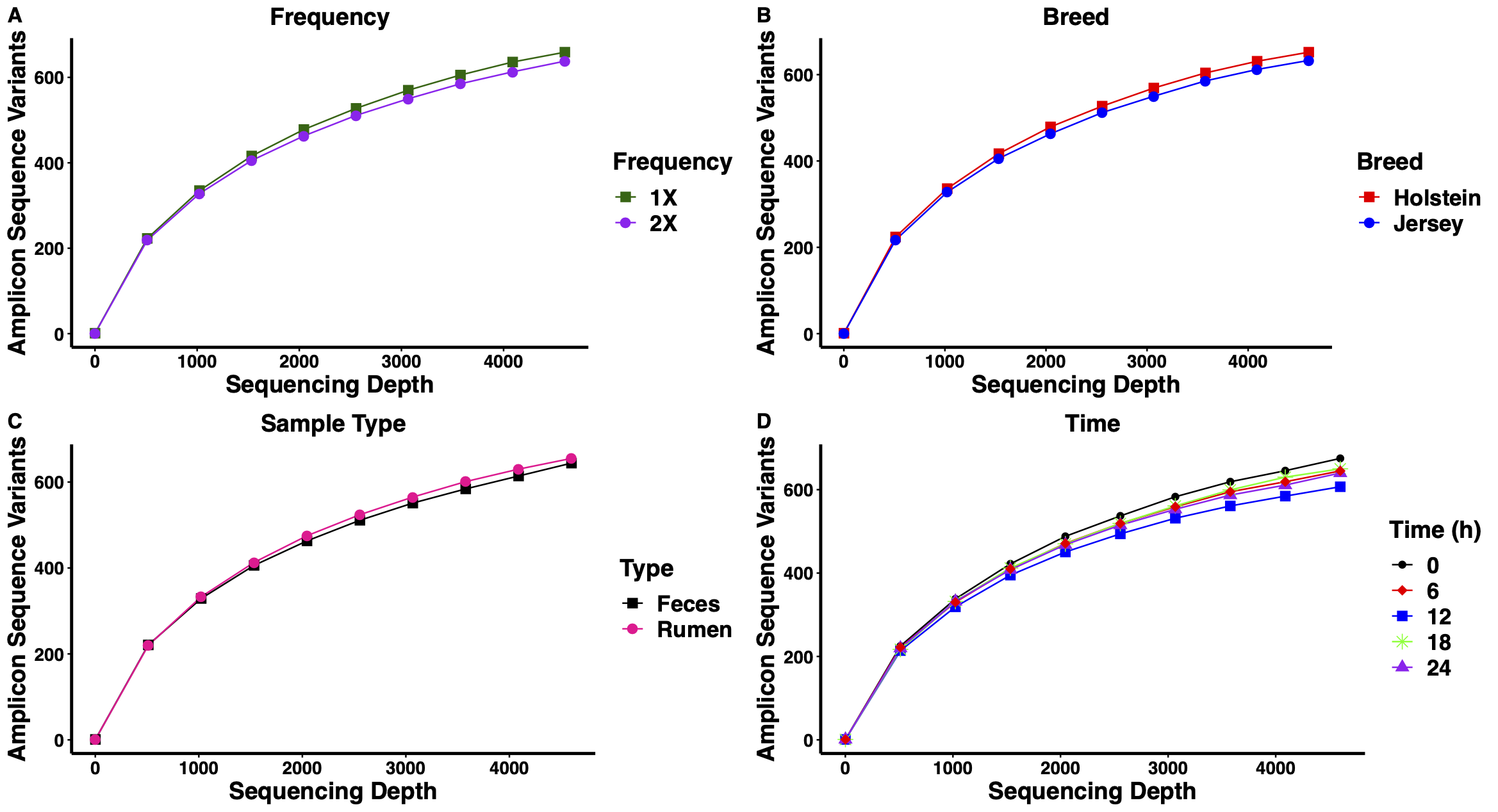

Supplement: S2 Fig — (A) Feeding frequency, (B) breed, (C) sample type, and (D) collection time (0 [pre-feeding am], 6, 12 [pre-feeding pm], 18, and 24 [pre-feeding am] h). Samples were rarefied at an even depth of 4,600 reads and values represent medians from 10 iterations. (TIF) [file pone.0248147.s002.tif]

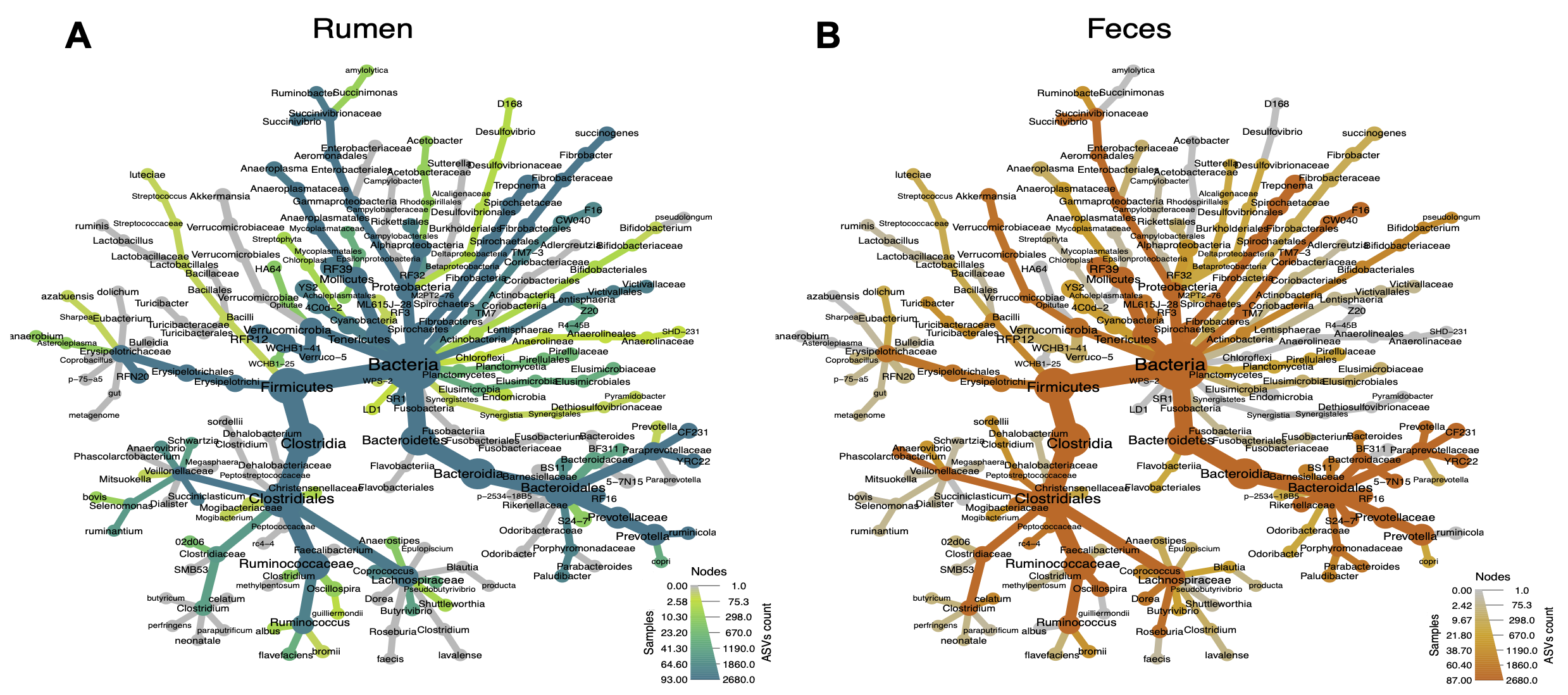

Supplement: S3 Fig — Color and size of the nodes (circles) and edges (lines) correspond to the relative abundance of the respective taxonomic rank. (TIF) [file pone.0248147.s003.tif]

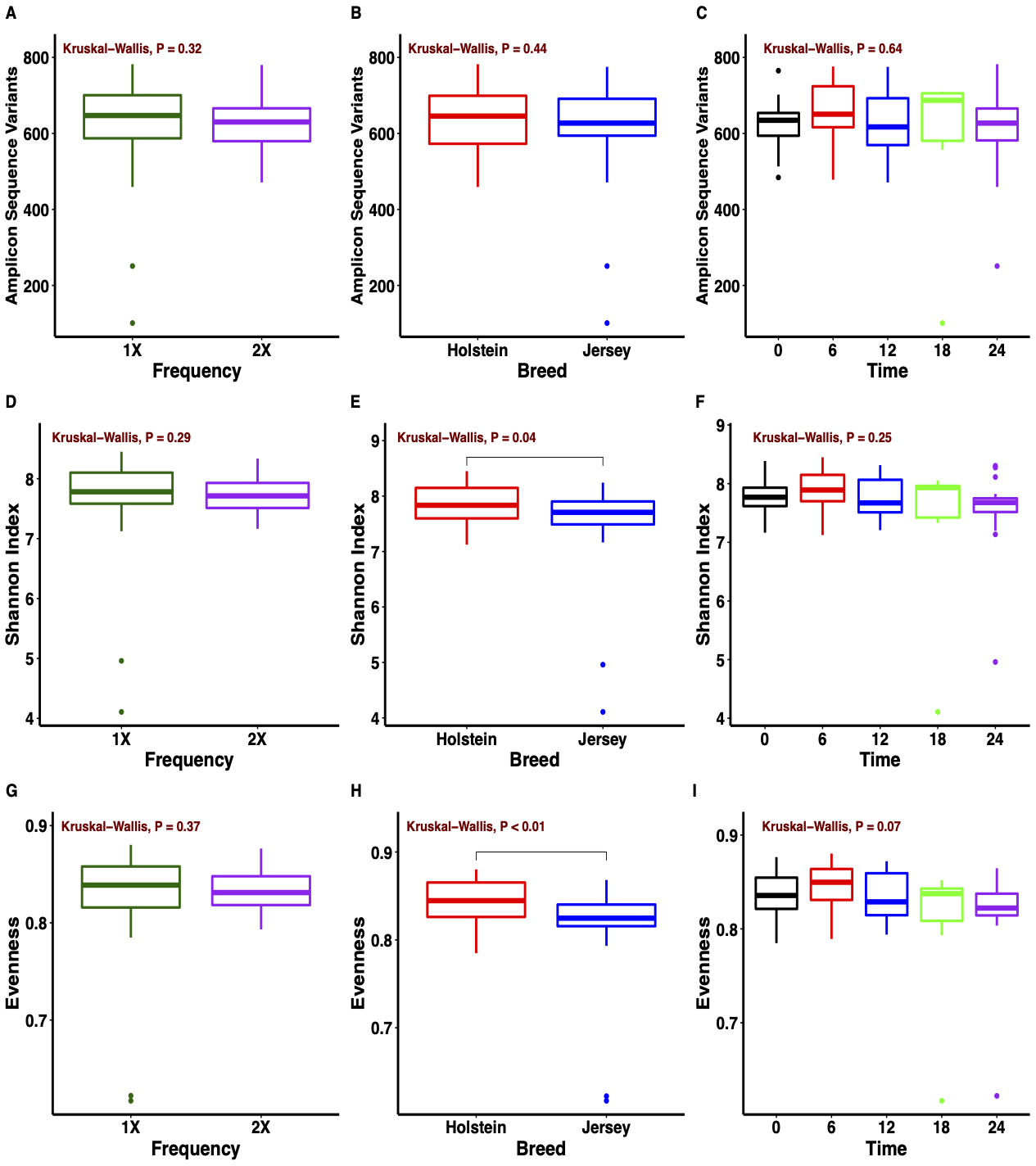

Supplement: S4 Fig — (A, B, C) Observed amplicon sequence variants, (D, E, F) Shannon index, and (G, H, I) evenness for feeding frequency, breed, and collection time (0 [pre-feeding am], 6, 12 [pre-feeding pm], 18, and 24 [pre-feeding am] h). (TIF) [file pone.0248147.s004.tif]

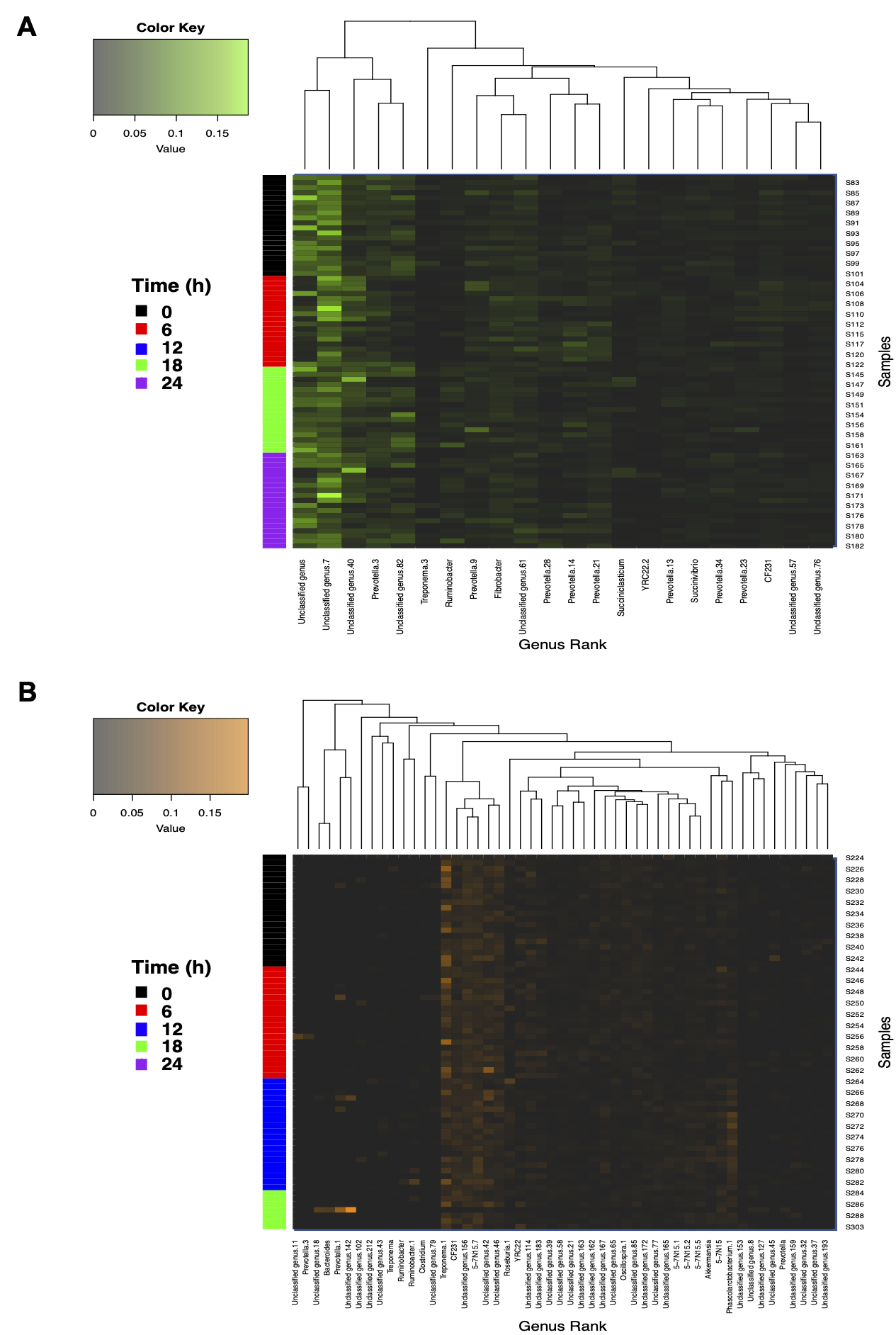

Supplement: S5 Fig — (A) Rumen and (B) fecal samples from both Holstein and Jersey cows. Amplicon sequence variants with a relative abundance > 1% are presented. Collection time = 0 (pre-feeding am), 6, 12 (pre-feeding pm), 18, and 24 (pre-feeding am) h. (TIF) [file pone.0248147.s005.tif]
