# Supplementary material for: Influence of industry standard feeding frequencies on behavioral patterns and rumen and fecal bacterial communities in Holstein and Jersey cows
Source: PLoS One. 2021 Mar 5;16(3):e0248147. doi: 10.1371/journal.pone.0248147 (PMC7935240; doi:10.1371/journal.pone.0248147)
Supplement: S1 Table — (DOCX) [file pone.0248147.s006.docx]

**S1 Table.** **Ingredient and chemical composition of the diet.**

| Item | % DM |
| --- | --- |
| Ingredient |  |
| Corn silage | 39.5 |
| Millet Baleage | 10.7 |
| Grass hay | 2.6 |
| Ground corn | 20.0 |
| Soybean meal | 10.8 |
| Distillers dried grains with solubles | 7.9 |
| Amino acid supplement^1^ | 1.9 |
| Rumen inert fat^2^ | 1.9 |
| Minerals and vitamins mix^3^ | 4.6 |
| Assayed composition |  |
| DM, % as fed | 41.0 |
| CP | 15.5 |
| NDF | 35.6 |
| Starch | 25.0 |
| Crude fat | 4.3 |
| Ash | 6.8 |
| NEL (Mcal/kg) | 1.67 |

^1^Pro-Team70 (Perdue Agribusiness, Salisbury, MD).

^2^Energy Booster 100 (Milk Specialties Global, Eden Prairie, MN).

^3^Contained 11.2% Ca, 0.4% P, 4.5% Mg, 4.9% K, 12.5% Na, 7.4% Cl, 1.1% S, 1918 mg of Mn/kg, 304 mg of Cu/kg, 1991 mg of Zn/kg, 20 mg of I/kg, 14 mg of Co/kg, 7 mg of Se/kg, 63,960 IU of vitamin A/kg, 16964 IU of vitamin D/kg, and 379 IU of vitamin E/kg.
