# Supplementary material for: Influence of industry standard feeding frequencies on behavioral patterns and rumen and fecal bacterial communities in Holstein and Jersey cows
Source: PLoS One. 2021 Mar 5;16(3):e0248147. doi: 10.1371/journal.pone.0248147 (PMC7935240; doi:10.1371/journal.pone.0248147)
Supplement: S2 Table — (DOCX) [file pone.0248147.s007.docx]

**S2 Table. Diurnal patterns of the rumen and fecal pH and rumen fermentation parameters.**

|  | Collection Time^1^ | | | | |  | *P*-value |
| --- | --- | --- | --- | --- | --- | --- | --- |
| Measure | 0 | 6 | 12 | 18 | 24 | SEM | T |
| Rumen pH | 6.63^ab^ | 6.50^c^ | 6.57^bc^ | 6.75^a^ | 6.75^a^ | 0.06 | <0.01 |
| Feces pH | 6.66^bc^ | 6.83^ab^ | 7.02^a^ | 6.65^bc^ | 6.63^c^ | 0.07 | <0.01 |
| Ammonia, mg/dL | 8.20 | 9.98 | 8.36 | 8.89 | 8.47 | 0.60 | 0.50 |
| Total VFA, m*M* | 228^a^ | 218^a^ | 140^b^ | 130^b^ | 151^b^ | 10.2 | <0.01 |
| VFA, mol/100 mol |  |  |  |  |  |  |  |
| Acetic acid | 72.2 | 69.8 | 69.6 | 69.9 | 71.0 | 1.40 | 0.12 |
| Propionic acid | 15.5 | 17.4 | 17.3 | 16.8 | 16.7 | 1.26 | 0.32 |
| Butyric acid | 11.2 | 11.5 | 11.9 | 12.1 | 11.2 | 0.40 | 0.23 |
| Valeric acid | 1.01 | 1.19 | 1.16 | 1.18 | 1.02 | 0.07 | 0.14 |

^a–c^Mean values in the same row with different superscripts differ.

^1^0 (pre-feeding am), 6, 12 (pre-feeding pm), 18, and 24 (pre-feeding am) h
